# Emerging Role of Vitamin D and its Associated Molecules in Pathways Related to Pathogenesis of Thrombosis

**DOI:** 10.3390/biom9110649

**Published:** 2019-10-24

**Authors:** Syed Mohammad, Aastha Mishra, Mohammad Zahid Ashraf

**Affiliations:** Department of Biotechnology, Faculty of Natural Sciences, Jamia Millia Islamia, New Delhi 110025, India; mahmudaga@gmail.com (S.M.); aastha0602@gmail.com (A.M.)

**Keywords:** vitamin D, thrombosis, coagulation pathway, inflammation, activated endothelial cells

## Abstract

Vitamin D, besides having an essential role in calcium and bone metabolism, also acts as a mediator of many non-calcemic effects through modulations of several biological responses. Vitamin D exists in its two major forms, vitamin D_2_, or commonly known as ergocalciferol, and vitamin D_3_, or commonly known as cholecalciferol. Both of these forms bind to vitamin D-binding protein to get transported to all vital target organs, where it serves as a natural ligand to vitamin D receptors for enabling their biological actions. Clinical reports corroborating vitamin D deficiency with an increase in thrombotic episodes implicate the role of vitamin D and its associated molecule in the regulation of thrombosis-related pathways. Thrombosis is the formation and propagation of a blood clot, known as thrombus. It can occur either in the arterial or the venous system resulting in many severe complications, including myocardial infarction, stroke, ischemia, and venous thromboembolism. Vitamin D, directly or indirectly, controls the expression of several genes responsible for the regulation of cellular proliferation, differentiation, apoptosis, and angiogenesis. All of these are the processes of potential relevance to thrombotic disorders. This review, thus, discussed the effects of vitamin D on pathways involved in thrombosis, such as hemostatic process, inflammatory pathway, and endothelial cell activation, with a focus on the molecular mechanisms associated with them.

## 1. Background

Vitamin D is a lipophilic, secosteroid hormone, which majorly exists in two forms—vitamin D_2_ and vitamin D_3_. Vitamin D_2_, commonly known as ergocalciferol, is manufactured through the ultraviolet irradiation of ergosterol from yeast [1]. It cannot be synthesized inside the human organism but can enter blood circulation with dietary sources, such as mushroom, supplementation, and fortification [2]. Vitamin D_3_, commonly known as cholecalciferol, is the only form produced in the human organism that is synthesized in the skin through the ultraviolet irradiation of 7-dehydrocholesterol. It can also be obtained from sources like fatty fish, egg and dairy products, and dietary supplements. In mammals, synthesis of vitamin D_3_ gets initiated in the epidermis, with cleavage of the B ring of 7-dehydrocholesterol, under UVB radiation of wavelength 290 to 315 nm. This gives rise to an extremely unstable previtamin D_3_, which upon spontaneous isomerization, is converted to a precursor molecule, vitamin D_3_ [3]. This is then converted to 25-hydroxy vitamin D_3_ (25(OH)D_3_) in the liver by enzyme 25-hydroxylase [4]. Similarly, vitamin D_2_ after ingestion undergoes a 25-hydroxylation step in the liver, yielding 25-hydroxy vitamin-D_2_ (25(OH)D_2_) [5]. Both 25(OH)D_2_ and 25(OH)D_3_ are further subjected to a second hydroxylation step in the kidney to produce hormonally active forms, 1,25-dihydroxyvitamin D_2_ (1,25(OH)_2_D_2_) and 1,25-dihydroxyvitamin D_3_ (1,25(OH)_2_D_3_), respectively [5]. 25-hydroxy vitamin D (25(OH)D), reflecting the concentrations of both 25(OH)D_2_ and 25(OH)D_3_, is routinely determined to assess vitamin D status in clinical practices [6]. It has been observed that vitamin D_3_ is more efficacious at raising concentrations of serum 25(OH)D than D_2_ [7,8]. This could be attributed to the fact that vitamin D_3_ has a higher affinity for vitamin D binding protein as well as for the vitamin D receptor and is also the preferred substrate for the hydroxylase enzyme in the liver in comparison to vitamin D_2_ [9]. Further, vitamin D_2_ is observed to be considerably less stable than vitamin D_3_ [10].

Vitamin D deficiency is a highly prevalent condition, affecting about 30–60% of the general population worldwide [11,12,13]. It is conventionally considered to be the main factor in skeletal homeostasis and in the prevention of rickets in children and osteomalacia and osteoporosis in adults [14,15,16,17]. In addition to its classical role in bone metabolism, it has been demonstrated that vitamin D is also a crucial agent for decreasing the risk of cardiovascular disorders (CVDs), malignancies, and immune system disease [18,19,20,21,22,23]. The significance of this biomolecule could be demonstrated by the fact that almost a 7% reduction in all-cause mortality can be attained by adequate serum vitamin D levels and are expected to ease the economic burden of diseases [24,25,26]. Epidemiological data indicate that deficiency of vitamin D is common among CVDs patients with circulating 25(OH)D levels less than 20 ng/mL [11]. Similarly, a reduced plasma level of 25(OH)D of around 25 ng/mL has been associated with an increased risk of hypertension [27,28]. A population-based study and meta-analyses associated very low levels of 3–4.8 ng/mL of plasma 25(OH)D levels with an increase in multivariable-adjusted risk by 40% for ischemic heart disease, by 64% for myocardial infarction (MI), and by 57% for early death when compared with the individuals having plasma 25(OH)D levels of 18.83–28.44 ng/mL [29]. All these data suggest that the deficiency of vitamin D levels is an alarming risk factor for such diseases. Table 1 provides the vitamin D status, viz. severely deficient, deficient, insufficient, and recommended level as per the serum levels of vitamin D in the body. The serum levels of vitamin D less than 20 ng/mL is considered to be deficient in the human body.

Vitamin D is a key metabolite with diverse metabolic processes in the human body. Its active forms bind to vitamin D binding protein (DBP) so that they get transported to all the vital target organs of the body. These active forms then serve as a natural ligand to vitamin D receptor (VDR) and bring about the biological functions of vitamin D [32,33]. VDR is a ligand-active transcription factor that plays a vital role in regulating the expression of several genes involved in calcium phosphate homeostasis [34]. VDR is distributed throughout the body in various cells and tissues, such as the epithelial cells of the small intestine, large intestine, distal tubule of kidneys, parathyroid, bronchus, thymus, prostate gland, mammary gland, endothelial cells, and the cells of the osteoblasts in bones [35,36,37]. VDR has also been identified in most of the immune cells, notably in macrophages, dendritic cells, and lymphocytes, and has shown to be involved in the regulation of inflammatory and immune responses [38,39,40,41]. Inflammation and hemostasis are the two complex pathophysiological processes that are interrelated to each other. The activation of both the systems is interdependent in a positive feedback loop with one process promoting the effects of the other process and vice versa. This crosstalk involves procoagulant factors, pro-inflammatory cytokines, chemokines, adhesion molecules, tissue factor (TF) expression, platelets, and endothelial cells [42]. These all mediators trigger the prothrombotic tendency by an increase of platelet reactivity, endothelial dysfunction, activation of the coagulation cascade, and suppression of natural anticoagulant pathways, as well as fibrinolytic activities [43]. Therefore, it becomes imperative to consider the influence of vitamin D and VDR in the thrombosis pathophysiology.

Thrombosis is the formation and propagation of a blood clot, known as a thrombus, in the either arterial or venous system. It generally occurs when there is a disturbance in the balance between clot formation and its dissolution [44]. Red blood cells and fibrins are the major contenders of the “red clot” seen in venous thrombosis, while aggregated platelets are the major contenders of the “white clot” seen in arterial thrombosis [45]. Virchow famously postulated three main causes of thrombosis: stasis of blood, change in the vessel wall, and change in the composition of blood [46]. Venous thrombosis has been associated with hypercoagulability (composition change) and reduced blood flow (stasis), whereas arterial thrombosis has been linked to endothelium disruption and platelet activation [47]. However, this traditional view of perceiving venous and arterial thrombosis as two different entities has been challenged recently. Patients with arterial thrombosis are also at the risk of developing venous thrombosis, and overlapping risk factors are associated with both arterial and venous thrombosis [48]. Furthermore, several novel factors have emerged in the pathogenesis of thrombosis. Plasma levels of vitamin D metabolites and its associated molecules are one of them that have appeared to be an intriguing factor involved in regulatory processes related to thrombosis [49]. There are shreds of evidence from the clinical reports that have correlated deficiency of vitamin D with the increase in thrombotic episodes [50,51]. Besides, Vitamin D, directly or indirectly, controls the expression of more than 200 genes, such as epidermal growth factor receptor, phospholipase C, gamma 1, Insulin growth factor binding protein 3, and many more, that are responsible for the regulation of cellular proliferation, differentiation, apoptosis, and angiogenesis [52]. These are the processes of potential relevance to thrombotic disorders. This review, therefore, tried to provide currently available data on the effects of vitamin D on coagulation factors, inflammatory cells, and endothelial cells that are involved in the thrombus formation with a special focus on their molecular mechanisms. We have also summarized recent progress related to favorable anti-thrombotic actions of vitamin D in tissues that are directly or indirectly involved in the thrombotic processes.

## 2. Role of Vitamin D in the Coagulation Pathway

Hemostasis is a dynamic process that maintains a balance between the clot formation and the mechanism that inhibits the clot [53]. The thrombogenic components of the body comprise of TF, prothrombin, fibrinogen, von Willebrand factor, collagen, platelet-activating factor, platelets, and activated endothelium. While the anti-thrombogenic components are antithrombin, plasminogen, tissue plasminogen activator, heparin, thrombomodulin, and protein C and S [53]. The anti-thrombotic effects of vitamin D on these thrombogenic and anti-thrombogenic components of the coagulation system have been very well documented [51,54,55]. A recent study demonstrated the association of low vitamin D levels with the development of deep venous thromboembolic (DVT) events in patients with ischemic stroke [51]. Likewise, a case-control study observed that the concentration of 25(OH)D was significantly lower in 82 participants with idiopathic lower-extremity DVT as compared to 85 sex and age-matched healthy participants [50]. Further, Blondon et al. [56] found a protective effect of vitamin D supplementation on the risk of unprovoked venous thromboembolism (VTE) and women with low vitamin D concentrations. This finding suggests that vitamin D deficiency could be treated as an undiagnosed risk factor for unprovoked VTE that constitutes around 40% cases of all VTE [56].

The effects of vitamin D metabolites through the noncalcemic actions of VDR ligands could be attributed to these antithrombotic effects of vitamin D [52]. A murine model-based study demonstrated the antithrombotic action of VDR through their VDR knock out (KO) mice fed either on high calcium or regular calcium diet. They found that platelet aggregation was enhanced and gene expression of antithrombin (AT) in the liver and thrombomodulin (TM) in the aorta, liver, and kidneys was downregulated in their VDR(KO) mice irrespective of their calcium diet. Whereas, *TF* expression in the liver and kidney was upregulated [57]. All of these results in their in vivo model elicit the role of VDR in the maintenance of antithrombotic homeostasis. Similarly, Ohsawa et al. [58] demonstrated the effects of 1,25(OH)_2_D_3_ and its potent synthetic analogs on *TF* and *TM* gene expressions when stimulated by tumor necrosis factor (TNF), lipopolysaccharides (LPS), and oxidized LDL (ox-LDL). They found that like 1,25(OH)_2_D_3_, its synthetic analogs downregulated *TF* and upregulated *TM* expressions, counteracting the effects of TNF and ox-LDL in monocytic cells. Perhaps, the biological activity of these analogs is mediated by VDR as these analogs have shown to have a higher affinity for VDR. These potent analogs may be further utilized for studying the molecular mechanisms of TF and TM regulation and might serve as adjunctive antithrombotic agents in the treatment of inflammatory and atherosclerotic diseases [58]. Similarly, Martinez-Moerno and colleagues investigated the impact of vitamin D molecules, calcitriol, and paricalcitol on the *TF* expression induced by a pro-inflammatory cytokine, TNF-α in human aortic vascular smooth muscle cells (VSMCs). Both calcitriol and paricalcitol, are a man-made active form of vitamin D. Their study demonstrated increased *TF* expression and procoagulant activity in a nuclear factor-κB (NF-κB) dependent manner in VSMCs incubated with TNF-α. This was accompanied by the upregulation of TF signaling mediator protease-activated receptor 2 (PAR-2) expression. However, upon treatment with calcitriol and paricalcitol, TNF-α-induced TF expression and activity associated with downregulation of NF-κB signaling and PAR-2 expression were significantly blunted, the levels of VDR were restored, and the expression of TF pathway inhibitor (TFPI) was enhanced [59]. An anti-coagulation protein, TFPI, acts as a dual inhibitor of coagulation by binding to both TF/Factor VIIa complex as well as Factor Xa [60]. One of the studies, in fact, found a significant positive association between more than 20 ng/mL levels of 25(OH)D_3_ and TFPI levels [61]. Furthermore, Toderici et al. described the transcriptional regulation of *AT* gene by vitamin D and described the functional and pathological relevance of mutations affecting the VDR elements of *AT* gene. Their study identified regulatory mutations in the *AT* gene resulting in AT deficiency and the risk of thrombosis. These regulatory mutations were found to disrupt potential VDR elements present in the promoter region of the gene, suggesting the crucial role of vitamin D on its regulation [62].

Furthermore, VDR has recently been found in human platelets and a megakaryocyte lineage [63]. Platelets are critically important mediators of the coagulation pathway, activating the TF-mediated processes and contact-arms of the coagulation cascade [64,65]. Larger platelets are shown to be more reactive and have greater pro-thrombotic potential. The elevated mean platelet volume (MPV) is associated with increased risk for DVT and MI in a large sample size study, supporting the concept that platelet reactivity is indispensable in the pathogenesis of venous thromboembolism [66]. Furthermore, a recent study has associated the increase in MPV with a decrease in serum 25(OH)D concentrations in patients with stable coronary artery disease, suggesting further linkage of vitamin D to thrombosis and hemostasis [55]. They concluded that there exists a negative correlation between MPV and serum 25(OH)D concentration, with MPV highest in group of patients with 25(OH)D concentration <10 ng/mL, moderate in group with the concentration of 10–20 ng/mL, and lowest in group with the concentration >20 ng/mL.

## 3. Role of Vitamin D in Inflammatory Pathways

Evidence of an intrinsic link between innate inflammatory system and coagulation challenges the definition of pathological thrombosis, as described by Virchow [46]. In 2013, Engelmann and Massberg coined the term immunothrombosis, describing a process by which the activation of coagulation assists the function of the innate immune system, and the converse, whereby components of the immune system contribute to thrombosis [67]. Recently, a study from our lab demonstrated the crucial role of sterile inflammation in the thrombotic events and also established that inflammation precedes coagulation in thrombosis [68]. Our study showed a direct association between nucleotide-binding domain, leucine-rich-containing family, pyrin domain containing 3 (NLRP3) inflammasome complex and hypoxia-inducible factor 1-alpha (HIF-1α) in hypoxia-induced thrombosis. We also observed a concomitant increase in the relative expression of NLRP3, caspase-1, interleukin-1β (IL-1β), and interleukin-18 (IL-18) transcripts in the individuals with clinically established venous thrombosis [68]. Additionally, Yadav et al. demonstrated the orchestration of venous thrombosis under normal oxygen concentration by the increased activation of NLRP3 inflammasome and IL-1β release in their CD39-deficient mice [69].

TF, a key initiator for in vivo activation of the extrinsic pathway of coagulation, is one of the links between inflammation and coagulation. Inflammatory mediators promote coagulation by elevating the production of TF. Normally, TF is present in the circulations but at a very low level; however, an increase of TF expression and its interaction with platelets and coagulation factors tend to shift the hemostatic balance in favor of coagulation or thrombosis [64,65]. Platelets store and release not just those biologically active substances required for coagulation and vascular integrity, but it also contains substances, including growth factors, cytokines, and chemokines, that affect immunological responses [70]. Platelets interact with neutrophils, monocytes, and lymphocytes to activate them and are simultaneously responsible for the initiation and amplification of venous thrombosis [71]. Functional Toll-like receptors (TLRs), such as TLR-2, TLR-4, and TLR-9, expressed on platelets also provide a potential link to innate immunity with thrombosis [72]. The multifaceted role of fibrinogen, an acute phase reactant, in tissue injury and inflammation also provides the connection between these two pathways. Studies have shown the deposition of fibrinogen in both inflammatory conditions and tissue injury. Thus, all of these points of commonality reflect the close connection of inflammation and thrombosis [73].

Vitamin D has been instrumental in the proliferation, differentiation, and functions of immune cells, both directly and indirectly. Dendritic cells and macrophages are known to express enzymes, 25-hydroxylase and 1-alpha-hydroxylase, required for converting vitamin D to its activated form, i.e., 1,25(OH)_2_D, whereas, activated T cells can hydrolyze 25(OH)D to 1,25(OH)_2_D [74,75,76]. A study demonstrated that vitamin D inhibited the production of pro-inflammatory cytokines, like IL-6 or TNF-α, in monocytes/macrophages via the inhibition of p38 MAP kinase [77]. Zhang et al. identified that a dose of vitamin D stimulated and upregulated the expression of MAPK phosphatase-1, which dephosphorylated p38 and inhibited p38 phosphorylation and inactivation, thereby inhibiting the production of pro-inflammatory cytokines, IL-6, and TNF-α production in LPS-stimulated human monocytes [77]. Another study demonstrated the profound anti-inflammatory effect of vitamin D on peripheral and intestinal T cells from inflammatory bowel disease patients. They incubated peripheral and intestinal T-cells with 1,25(OH)_2_D that resulted in a significant reduction in frequencies of pro-inflammatory T cells producing IFN-γ, IL-17, IL-22, IL-9, and TNF [78]. Furthermore, 1,25(OH)2D_3_ has been reported to decrease the expression of transcription factors, NF-κB, leading to an anti-inflammatory effect [79].

## 4. Role of Vitamin D in the Endothelial Cell Activation

The vascular endothelium is located strategically at the interface between tissue and blood, which is an ideal position, to modulate the entire cardiovascular system. Endothelial cells (ECs) play an essential role in the maintenance of vascular tone, transport of nutrients and solutes across the endothelium. It also plays an active role in the maintenance of a thrombo-resistant surface through the activation and inactivation of various vasoactive hormones [80]. Under normal conditions, ECs provide a non-thrombogenic surface, which prevents the adhesion of the platelets or other blood cells and does not activate the coagulation cascade [81]. Under physiological conditions, the anticoagulant role of ECs is largely mediated by TFPI, thrombomodulin, endothelial progenitor cell receptor, heparin-like proteoglycans, and platelet inhibitors, such as nitric oxide and prostacyclin. However, once ECs become activated, they become pro-thrombotic. For example, activated endothelial cells express TF and play an active role in the generation of thrombin [82]. Similarly, activated ECs also express a variety of molecules, such as von Willebrand factor, P-selectin, angiopoietin-2, and endothelin-1 (ET-1), which are the active participants of a variety of processes, such as platelet adhesion, leukocyte recruitment, inflammation modulation, and vasoconstrictor [83,84].

Few interventional studies have demonstrated the improvement in endothelial functions on treatment with vitamin D [85,86]. Studies suggest that vitamin D improves endothelial function [87,88] and reduces the production of inflammatory cytokines [89]. It may also cause a decrease in endothelial adhesion molecules, an increase in NO production, and the reduction of platelet aggregation [57,90,91]. Other potential mechanisms linking vitamin D to vascular health include the decrease in oxidative stress [89] and attenuation of NF-κB activation [92]. NF-κB signaling molecules regulate the activation of ECs by giving rise to a pro-adhesive and pro-coagulant phenotype with a concomitant reduction of the ECs barrier function [93]. The key target genes of NF-κB in the ECs include adhesion molecules, such as vascular cell adhesion molecule 1 (VCAM-1), intercellular adhesion molecules 1 (ICAM-1), and E-selectin that mediates adherence of inflammatory cells, including neutrophils, monocytes, lymphocytes, and monocytes, to the vascular wall and triggering their extravasation into tissue [94,95,96,97].

Expression of VDR and its ligand-generating enzyme, 1-alpha-hydroxylase, in ECs [98] suggests that they possess the capability for orchestrating local vitamin D dependent regulatory activity that is confined to a vascular cell. Furthermore, vitamin D stimulates the production of vasoactive factors in human ECs culture [99]. Calcitriol upregulated the ET-1 and NO production via VDR-dependent activation as the effects were shown to get disappeared when the VDR gene was silenced. However, the mechanism involved in the upregulation of each factor differed. While ET-1 upregulation was regulated by activator protein-1 (AP-1) activation, the upregulation of NO via endothelial nitric oxide synthase (eNOS) was controlled directly by VDR activation. This group also evaluated the in vivo consequence of injecting a single intraperitoneal injection of 400 ng/kg calcitriol in normal rats and euthanized them 24 h later. Calcitriol increased the production and expression of both factors in the rats, confirming the results observed in the endothelial cell culture [99]. A study showed that even a presumed inactive sterol, cholecalciferol, is a potent mediator of endothelial stability at physiologically relevant levels in a VDR-independent manner. This research study found that the stabilizing effect was non-genomic and occurred in conjunction with the deactivation of ADP-ribosylation factor-6 (ARF-6), RhoA deactivation, and stabilization of vascular endothelial-cadherin at the plasma membrane [100].

In another study, expression of eNOS was shown to be reduced by 50% in aortic tissue of VDR gene knockout when compared with the wild type mice [54]. A research paper employing an endothelial-specific knockout of the murine *VDR* gene showed a significant impairment in acetylcholine-induced aortic relaxation [101]. This was accompanied by a reduction in eNOS expression and phosphor-vasodilator-stimulated phosphoprotein levels in the aorta as compared to control mice [101]. Furthermore, brachial artery flow-mediated dilation, a measure of endothelium-dependent dilation in vivo, was lower in vitamin D insufficient group. In this study, flow-mediated dilation was positively related to serum 25(OH)D but not 1,25(OH)2D concentration. They also observed that vascular ECs expression of p65 subunit of NF-κB, pro-inflammatory nuclear transcription factor, and IL-6, a pro-inflammatory cytokine downstream target of NF-κB, was greater in vitamin D deficient subjects when compared to their vitamin D sufficient subjects [93].

Suppression of the rennin-angiotensin-aldosterone system (RAAS) and positive effects on endothelial function [87] and vascular structure [102] are reported among the most reliable mechanisms explaining the influence of vitamin D on the cardiovascular system. The suppression of RAAS by VDR activation has been documented by using in vitro experiments [103] and animal models [104]. Human observational data confirm this inverse relation between RAAS parameters and 25(OH)D levels [105,106]. A study on thirty-three consecutive patients with essential hypertension and hypovitaminosis D underwent therapy with cholecalciferol for 8 weeks. At the end of the study, vitamin D levels were found to be normal, and the restored vitamin D levels inhibited peripheral RAAS system with the improvement in flow-mediated dilation in patients with essential hypertension and hypovitaminosis D [107].

Wei Xu et al. studied the effect of vitamin D on endothelial progenitor cells (EPCs) pretreated with AngII. Their study demonstrated that vitamin D reversed AngII-induced oxidative stress injury by the peroxisome proliferator-activated receptor-γ (PPAR-γ) pathway [108]. PPAR-γ lowers the production of superoxide and enhances the repair capacity of EPCs [109]. It also protects the EPC function against oxidative stress [110]. Ang II induces the production of reactive oxygen species and activates the transcription factor NF-κB. This induces several cytokines, such as TNF-α, IL-6, ICAM-1, VCAM-1, and E-selectin. The induction of these cytokines prompts vascular injury [111]. Schröder and group investigated the endothelial barrier characteristics of endothelial colony-forming cells (ECFCs) and the effect of vitamin D on cell-cell adhesion and barrier integrity of ECFCs monolayer under inflammatory conditions. ECFCs are a specific and well-defined sub-population of EPCs, participating in endothelial repair with the high proliferative ability of de novo vessel formation. Their study showed that Vitamin D treatment enhanced ECFC mobilization through vascular endothelial cadherin phosphorylation. Vitamin D treatment alleviated inflammation in ECFCs monolayer that resulted in higher ECFC barrier integrity [112]. All of these studies suggest the significant role of vitamin D on endothelial functions and its deficiency in the dysfunctioning of these cells.

## 5. Anti-Thrombotic Actions of Vitamin D

Several studies have highlighted the anti-thrombotic actions of vitamin D [51,54,55]. A pilot randomized control trial was done on forty vitamin D deficient patients with DVT or pulmonary embolism (PE) [113]. After three months of treatment with vitamin D supplementation, the researchers found enhancement in the anticoagulant effect of warfarin, i.e., those patients who were given vitamin D used significantly lower doses of warfarin as compared to the placebo. Another study demonstrated that cholecalciferol supplementation in stable, moderate chronic kidney disease patients was associated with an improved vascular endothelial function. Supplementation of vitamin D resulted in a significant decrease in parathyroid levels and also reduced the levels of circulating biomarkers of endothelial function, including E-selectin, VCAM, and ICAM-1 [114].

A prospective study on a cohort comprising of 40,000 women drawn from the southern Swedish population followed for a mean period of 11 years concluded that women with a habit of more active sun exposure were at a 30% lower risk of VTE than those who did not [115]. Women exposing themselves to the sun or artificial UVB light more often, presumably improve their vitamin D status [116]. Moreover, the risk of VTE increases by 50% in winter, during which vitamin D has been demonstrated to be the lowest [117], as compared to the other seasons.

Furthermore, a randomized controlled study of 250 patients with prostate cancer, half of whom were given 45 micrograms of 1,25(OH)_2_D_3_ weekly, found an unexpected significantly lower risk of thrombotic events [118]. A double-blind, placebo-controlled randomized trial in Scotland showed significant improvement in flow-mediated vasodilation of brachial artery and also decreased blood pressure with a single dose of 100,000 IU vitamin D_2_ to patients with type-2 diabetes [85]. However, few studies did not find any effect of vitamin D supplementation [117,118]. An interventional pilot study screened 76 Caucasian patients with peripheral artery disease for vitamin D deficiency and found that vitamin D supplementation increased serum 25-(OH)D levels without influencing endothelial function, arterial stiffness, coagulation parameters, such as thrombin, antithrombin, d-dimer, plasminogen activator inhibitor-1, and inflammation parameters, such as c reactive protein [119]. Another study found no effect on the improvement of markers of vascular function, such as blood pressure and total cholesterol with vitamin D supplementations, in patients with a history of MI [120].

## 6. Future Perspectives

Vitamin D has established itself as a key regulatory molecule in normal physiological processes and has shown tremendous promise as a predictive/therapeutic tool. There are ample studies, correlating lower doses of vitamin D with thrombosis [115,116,117,118]; however, the exact molecular mechanism of the pathophysiological role of lower doses of vitamin D is still obscure. It is now obvious that vitamin D has several non-calcemic consequences, which, when investigated, could lead us to the genesis of thrombosis that remains a conundrum to the scientific community. Vitamin D is critical at high altitudes since its levels are usually found to be drastically low as a consequence of the surrounding environment, such as excessive clothing due to extreme cold and harsh sunlight [121]. Furthermore, hypoxic environment facilitates the pro-thrombotic environment [68,122]. Therefore, it would be really interesting to hypothesize that exposure to high altitude hypoxia might perturb the expression and physiology of vitamin D, which in turn potentiates the onset of prothrombotic phenotypes via activation of NLRP3 inflammasomes-mediated inflammation. The conductance of such studies analyzing the differential plasma concentration of vitamin D and associated molecules at high altitude and their association with thrombotic episodes would add a new dimension to the thrombosis biomarker discovery. It would also provide the clue towards the integrated involvement of hypoxia-vitamin D-NLRP3 inflammasome triad contribution to the underlying mechanisms of vascular dysfunction/hypercoagulation under hypoxia. Our lab has conducted a preliminary unpublished study that observed a substantial effect of hypobaric hypoxia exposure on vitamin D concentration in both healthy persons and thrombotic patients at high-altitude. Our study observed that the changes in vitamin D concentration in thrombotic patients were much severe as compared to healthy exposed persons at high altitudes. The extension of such a study can also explore the potential of vitamin D to be used as therapeutic interventions for the efficient prevention of hypoxia-induced thrombotic disorders. To conclude, the understanding of vitamin D expression behavior under hypoxia and advances in the implication of the understanding in the context of thrombosis would help us in the translational application that could be beneficial in the prevention of several other lethal cardiovascular disorders.

## Figures and Tables

**Table 1:** Status of vitamin D level as provided by the Institute of Medicine* and Endocrine Society**.

Vitamin D Status	Institute of Medicine	Endocrine Society
Severely deficient	0–10 ng/mL	0–10 ng/mL
Deficient	-	10–20 ng/mL
Insufficient	10–20 ng/mL	20–30 ng/mL
Recommended level	20–50 ng/mL	30–100 ng/mL

* Institute of Medicine is a non-profit organization established by National Academy for science, USA, for science-based advice on biomedical science, medicine, and health [30]. ** Endocrine Society is a professional, international medical organization, USA, in the fields of endocrinology and metabolism [31].
